# Nuciferine inhibits osteoclast formation through suppressing glycolysis metabolic programming and ROS production

**DOI:** 10.1002/kjm2.12906

**Published:** 2024-11-16

**Authors:** Wen‐Hui Guo, Guan‐Qi Zhen, Feng Wu, Yun‐Peng Lv, Jing‐Long Yan, Jia‐Ning Zu, Cheng‐Chao Song

**Affiliations:** ^1^ Department of Orthopedics The Second Affiliated Hospital of Harbin Medical University Harbin China

**Keywords:** glycolysis, Nuciferine, osteoclast, ROS

## Abstract

Nuciferine (NCF) is a bioactive compound from lotus leaves and has been proven to prevent osteoclastogenesis and ovariectomy‐induced osteoporosis by our previous research. However, the underlying mechanism is still unclear. In this research, Raw264.7 cells were induced into osteoclast with or without NCF. CCK‐8 and Edu assays were performed to detect the effects of 30 μM NCF on cell viability and proliferation. TRAP staining and bone resorption assays were performed to observe the role of NCF in osteoclastogenesis and bone resorption. RT‐PCR and Western blot were performed to detect the effects of NCF on osteoclast‐related genes, glycolysis‐related genes, and reactive oxygen species (ROS)‐related genes. Seahorse assays, lactate concentration and glucose consumption were performed to observe cell metabolism change. DCFH‐DA fluorescent probe was used to detect ROS level. In this work, 30 μM NCF could not influence cell viability and cell proliferation. Osteoclast differentiation could be inhibited by 30 μM NCF. Bone resorption assay could also observe that bone resorption ability was successfully inhibited by 30 μM NCF. In seahorse assay, we discovered that NCF could decrease extracellular acid rate and increase oxygen consumption. RT‐PCR and Western blot results showed that NCF could decrease the expression of hexokinase2, pyruvate kinase muscle 2, and lactate dehydrogenase A and that NCF could also weaken the concentration of lactate. However, pyruvate kinase muscle 2 activator (GC69716) and lactate addition could promote osteoclastogenesis and bone resorption and promote the expression of c‐Fos and nuclear factor of activated T cells c1. Besides, NCF could also inhibit the production of ROS. In conclusion, NCF might inhibit osteoclast formation through inhibiting glycolysis metabolism and ROS production.

## 1 INTRODUCTION

Osteoporosis is a common skeletal disease characterized with destroyed bone microstructure, decreased bone strength, and increased bone fragility. The imbalance between bone formation and bone resorption is the fundamental pathological mechanism of osteoporosis.^1^ Compared to weakened bone formation, the relative hyperactive bone resorption caused by osteoclast attracts more attention in the prevention and treatment of osteoporosis.^2^ Based on the pathological mechanism, many drugs have been developed and applied clinically, such as denosumab and bisphosphonates. Although these drugs have achieved encouraging clinical efficacy, long‐term use of these anti‐osteoporotic drugs has been reported to result in some side effects like jaw osteonecrosis and atypical femoral fractures.^3^ Recently, because of their great potential in the treatment of various diseases, including osteoporosis, natural products arouse much exploration. In our previous research, we found that Nuciferine (NCF), a bioactive compound from lotus leaves, could inhibit the formation of osteoclast and prevent ovariectomy‐induced osteoporosis, which means that NCF might be a potential anti‐osteoporosis drug.^4^ However, its unique anti‐osteoporosis mechanism is not clear. Based on previous research, we intended to clarify the mechanism of NCF preventing osteoporosis.

Glycolysis is very important for osteoclast formation from monocyte/macrophage.^5^ Once receptor activator of nuclear factor κb (RANK) is stimulated by receptor activator of nuclear factor κb ligand (RANKL), increased glucose consumption could be observed, which means that glycolysis is activated during osteoclastogenesis. And glycolysis is the preferential metabolic mode during the process of bone resorption.^6^ During glycolysis, glucose is converted into pyruvate and then pyruvate is metabolized into lactate. Some researchers have reported that inhibition of glycolysis can efficiently suppress the formation and bone resorption of osteoclast and prevent the development of osteoporosis.^6^, ^7^ Besides, in the osteoclast culture media, increased content of lactate is detected.^8^ As more has been reported, lactate is not only a byproduct of metabolism but also functions as a signal messenger between cells.^9^ Lactate can attract the accumulation of osteoclast,^10^ and the bone resorption effects of osteoclast is heavily dependent on lactate.^11^ Furthermore, lactate can activate the production of reactive oxygen species (ROS),^12^ one of the important signaling molecule in the process of osteoclast formation. These reports suggested that glycolysis is very important for osteoclast formation. However, weather NCF influences glycolysis during osteoclastogenesis is not clear.

It has been widely accepted that NCF exerts anti‐inflammatory and antioxidative effects.^13^ In this research, we found that NCF inhibited the increased glycolysis caused by RANKL stimulation during the osteoclast differentiation and decreased the level of lactate and ROS. Addition of lactate could restore the decreased formation of osteoclast and bone pit area caused by NCF treatment. And the inhibition of glycolysis might be the mechanism of NCF in the osteoclast differentiation.

## 2 MATERIALS AND METHODS

### 2.1 Reagents

As previously described,^4^ NCF (475‐83‐2) was obtained from Sigma‐Aldrich. Murine RANKL (315‐11) was obtained from PeproTech. The first antibodies of c‐Fos (ab208942), hexokinase2 (HK2) (ab209847), NADPH Oxidase 1 (Nox1) (ab131088), TNF receptor‐associated factor 6 (TRAF6) (ab40675), heme oxygenase 1 (HO‐1) (ab68477), glutathione *S*‐reductase (GSR) (ab124995), and nuclear factor of activated T cells c1 (NFATc1) (ab25916) were obtained from Abcam. Active Rac1 Detection Kit (8815), the first antibodies of pyruvate kinase muscle 2 (PKM2) (4053), lactate dehydrogenase A (LDHA) (2012), and the second antibodies (4967) were all obtained from CST. Raw264.7 cells were obtained from the American Type Culture Collection; these cells were cultured and induced into osteoclast as we previously described.^14^ CCK‐8 (CK04) was purchased from Dojindo. TRAP staining kit (387A) was purchased from Sigma. Transcriptor First Strand cDNA Synthesis Kit (11483188001) and FastStart Universal SYBR Green Master Kit (04913850001) were purchased from Roche Diagnstic. XF96 analyzer (S7855A) was purchased from Agilent technology. Lactate content detection kit (BC2235) and DCFH‐DA ROS fluorescent probe kit (D6470) were all purchased from Beijing Solarbio Science & Technology. Glucose uptake assay kit (ab136955) was obtained from Abcam.

### 2.2 Cell viability assay

2 × 10^3^ Raw264.7 cells were planted in each well of 96‐well plate overnight and then 30 μM NCF was added with 100 μL culture medium (H‐DMEM medium containing 10% FBS) for 24, 48, and 72 h. At the indicated time, 10 μL of CCK‐8 regent was added and incubated at 37°C and an enzyme marker (GloMax®‐Multi Detection System) was used to test cell viability.

### 2.3 TRAP staining

To observe osteoclast, TRAP staining was performed, and the process was performed as we previously described.^14^ In brief, Raw264.7 cells (6.25 × 10^3^ cells/cm^2^) were planted in H‐DMEM media with 10% FBS overnight and then the culture media was discarded. After that, these cells were incubated in osteoclast differentiation medium (H‐DMEM media containing 10% FBS and 100 ng/mL RANKL) for 5 days to induce osteoclast formation, and the osteoclast differentiation medium was replaced every 2 days. To observe the influence of NCF in osteoclast formation, 30 μM NCF was added. After 5 days of culture, cell culture medium was discarded and cells were fixed by the fixation solution for 30 s and then cleaned by water for three times. After that, cells were stained by dyeing solution until the multinuclear osteoclasts were dyed satisfyingly with the observation under microscope (DM IL LED, Leica Microsystems).

### 2.4 Bone resorption

To observe the bone resorption ability of osteoclast, bone pit assay was performed. Raw264.7 (6.25 × 10^3^ cells/cm^2^) cells were planted on 24‐well osteoassay plates (Corning) and induced into osteoclast differentiation with or without NCF treatment for 5 days. Then, these cells were removed and the bone pit area was observed.

### 2.5 RT‐PCR

RT‐PCR assay was performed as we previously reported.^4^ In brief, 2 × 10^4^ Raw264.7 cells were planted in 24‐well plate and incubated with or without NCF in osteoclast‐induced differentiation medium for 72 h and then total RNA was collected with TRIzol reagent. cDNA was synthesized by a commercial kit from 2 μg RNA. mRNAs were detected by RT‐PCR using a FastStart Universal SYBR Green Master Kit, and their relative expression was normalized to β‐actin. The primer sequences were as follows.

**Table:** 

NFATc1: forward	5′‐CAGTGTGACCGAAGATACCTGG‐3′
Reverse	5′‐TCGAGACTTGATAGGGACCCC‐3′
c‐Fos: forward	5′‐CGGGTTTCAACGCCGACTA‐3′
Reverse	5′‐TTGGCACTAGAGACGGACAGA‐3′
HK‐2: forward	5′‐ATTGTGGCTGTGGTGAA −3′
Reverse	5′‐AATGTGACGCATCTCCTC‐3′
PKM2: forward	5′‐CCTTCAGGAAGACAGCCAAG‐3′
Reverse	5′‐AGTGCTGCCTGGAATCCTCT‐3′
LDHA: forward	5′‐CATTGTCAAGTACAGTCCACACT‐3′
Reverse	5′‐TTCCAATTACTCGGTTTTTGGGA‐3′
β‐actin: forward	5′‐AGAGGGAAATCGTGCGTGAC‐3′
Reverse	5′‐CTCGTTGCCAATAGTGATGACC‐3′

### 2.6 Western blot

Cell proteins were extracted as previously described. In brief, cells were lysed by RIPA buffer containing PMSF and protease inhibitor. And then, 30 μg proteins were separated on the SDS–PAGE gel and transferred to PVDF membrane. The PVDF membranes were blocked in milk for 2 h and incubated with first antibody overnight at 4°C. After the first antibodies were discarded, the membrane was washed for three times at room temperature. And then, the second antibodies were added and incubated at room temperature for 2 h. After the membrane was washed for three times, ECL solution (abs9434, Abisin) was added and the indicated proteins were observed.

### 2.7 Cell metabolism detection

XF96 analyzer was used to detect the metabolism shift after RANKL stimulation with or without NCF treatment. The procedures were operated according to the instructions. In brief, 2 × 10^3^ Raw264.7 cells were planted on CellTak 96‐well plate and centrifuged to the plate bottom. Then, cells were incubated with or without NCF in osteoclast‐induced differentiation medium for 72 h. Finally, 1.5 mM oligomycin, 1.0 mM FCCP, 1 mM rotenone, and 1.8 mM antimycin A were sequentially added to measure oxygen consumption rate, and 10 mM glucose, 2 mM oligomycin, and 50 mM 2‐deoxy‐D‐glucose were added at the indicated time to measure the extracellular acidification rate (ECAR).

### 2.8 Detection of lactate

The concentration of lactate in cell culture medium was detected by a commercial lactate content detection kit according to the instructions. In brief, 2 × 10^4^ Raw264.7 cells were planted in 24‐well plate and incubated with or without NCF in osteoclast‐induced differentiation medium for 72 h. Then, cell culture supernatant was extracted and 100 μL of cell culture supernatant was mixed with extraction solution I and centrifuged to obtain supernatant. And then, the supernatant was mixed with extraction solution II and followed by centrifugation to obtain supernatant which was used to detect the concentration of lactate with enzyme marker.

### 2.9 Detection of glucose uptake

The consumption of glucose was detected according to the instructions of glucose uptake assay kit. In brief, 2 × 10^3^ Raw264.7 cells were planted in 96‐well plate and treated with or without NCF for 72 h after RANKL was added. Then, these cells were starved and treated with 2‐DG and continuously cultured for 20 min. After removing culture medium, cells were lysed and centrifuged to collect supernatant. Reaction mix A, extraction buffer, neutralizing buffer, and reaction mix B were added successively. Enzyme marker was used to analyze the consumption of glucose.

### 2.10 Intracellular ROS detection

The intracellular ROS levels were detected according to a commercial DCFH‐DA ROS fluorescent probe kit. In brief, 2 × 10^3^ cells were planted into a 96‐well pate with or without NCF in osteoclast‐induced differentiation medium for 72 h. Then, the medium was discarded and the dye working solution was added. After 60 min of incubation, the working solution was removed and the cells were washed with pre‐warmed culture medium. The fluorescence intensity was observed under a fluorescence microscope (SP8, Leica Microsystems) to detect the ROS level.

### 2.11 Statistical analysis

All experiments were repeated at least three times. Data were represented as the mean ± standard deviation and analyzed by unpaired Student's *t*‐test or one‐way ANOVA using SPSS 26.0 software (IBM). *p < 0.05, **p < 0.01, and ***p < 0.001 were considered as statistical significance.

## 3 RESULTS

### 3.1 NCF inhibits osteoclast differentiation of Raw264.7 cells

**FIGURE 1:**
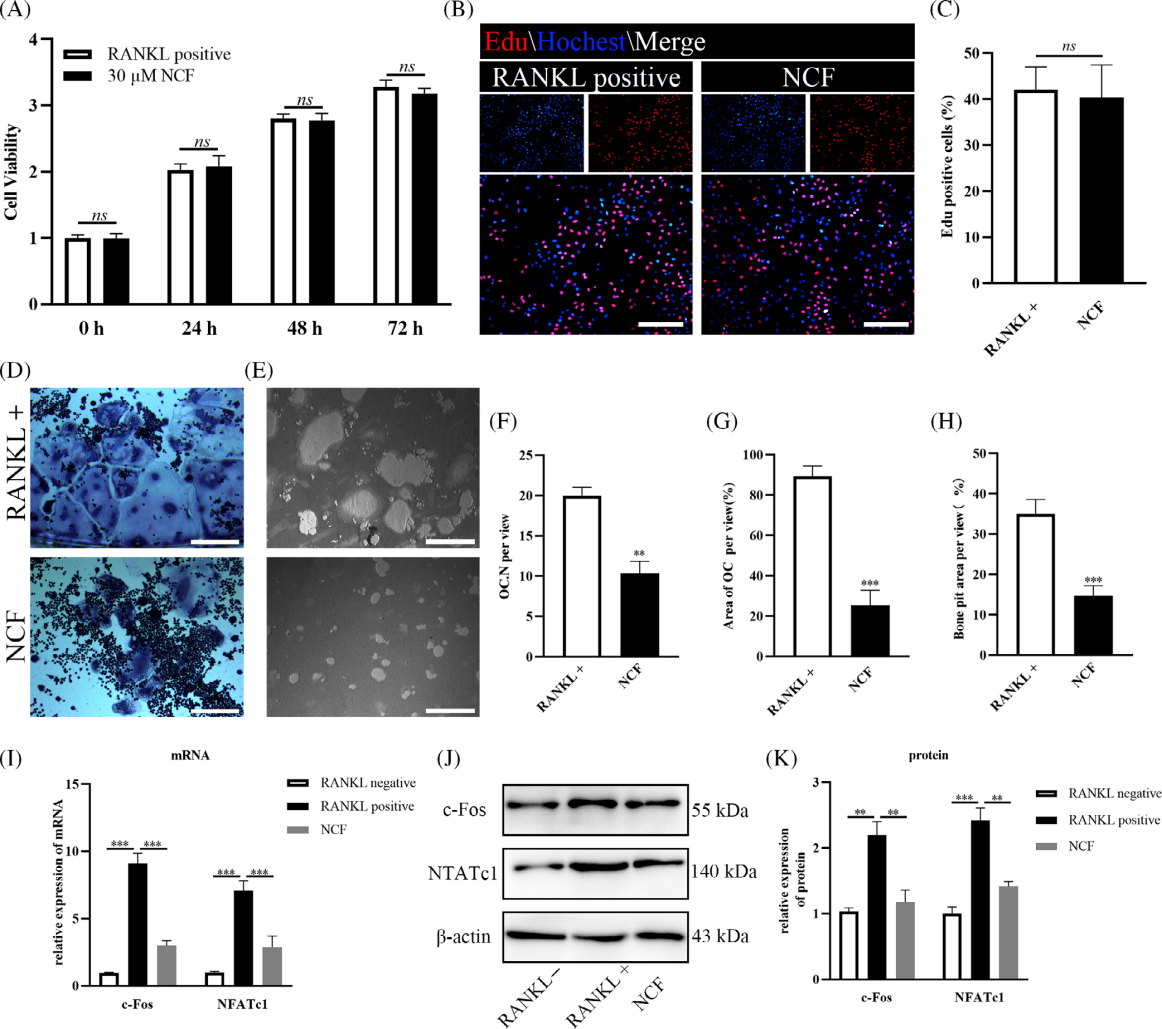
Thirty micromolar Nuciferine (NCF) inhibits osteoclastogenesis without influencing Raw264.7 cell's viability and proliferation. (A) Raw264.7 cells were treated with (NCF group) or without (RANKL positive) 30 μM Nuciferine for 24, 48, and 72 h, and CCK‐8 assay was performed to test cell viability. (B) Raw264.7 cells were treated with or without 30 μM Nuciferine for 72 h, and Edu assay was performed to test cell proliferation. Red indicated Edu‐positive cells, and nucleus was stained blue with Hochest. Scale bar = 100 μm (C) Quantitative analysis of Edu‐positive cells. (D) Raw264.7 cells were induced into osteoclast differentiation with or without NCF treatment for 7 days, and osteoclasts were multi‐nucleus TRAP‐positive cells. Scale bar = 200 μm (E) Raw264.7 cells were induced into osteoclast on osteoassay plates with or without NCF treatment for 7 days, and bone resorption was observed. Scale bar = 400 μm (F) Quantitative analysis of osteoclast number per view. (G) Quantitative analysis of area covered by osteoclast per view. (H) Quantitative analysis of bone resorption area. (I) The relative expression of c‐Fos and NFATc1 mRNA was examined by RT‐PCR. (J) The relative expression of c‐Fos and NFATc1 protein was detected by Western blot. (K) Quantitative analysis of the expression of c‐Fos and NFATc1 protein (**p < 0.01; ***p < 0.001). NFATc1, nuclear factor of activated T cells c1; RANKL, receptor activator of nuclear factor κb ligand.

According to our previous research,^4^ 30 μM NCF was chosen in this research. CCK‐8 assay showed Raw264.7 cells viability was not influenced by 30 μM NCF (Figure 1A). And Edu experiments also demonstrated that 30 μM NCF did not affect the proliferation ability of Raw264.7 cells (Figure 1B,C). TRAP staining was performed to indicate that the osteoclast differentiation of Raw264.7 cells was successfully inhibited by 30 μM NCF (Figure 1D). Thirty micromolar NCF could decrease the number of TRAP‐positive osteoclast (Figure 1F) and also weaken the area covered by osteoclast (Figure 1G). And bone resorption assay also displayed that 30 μM could inhibit bone pit area (Figure 1E,H), which means that 30 μM NCF could suppress the bone resorption ability of osteoclast differentiated from Raw264.7 cells. RT‐PCR and Western blot prompted that 30 μM could decrease the expression of c‐Fos and NFATc1 in Raw264.7 cell (Figure 1I–K), which play key roles in the osteoclast differentiation induced by RANKL.

### 3.2 NCF reshapes metabolic programming in Raw264.7 cells during osteoclast differentiation

**FIGURE 2:**
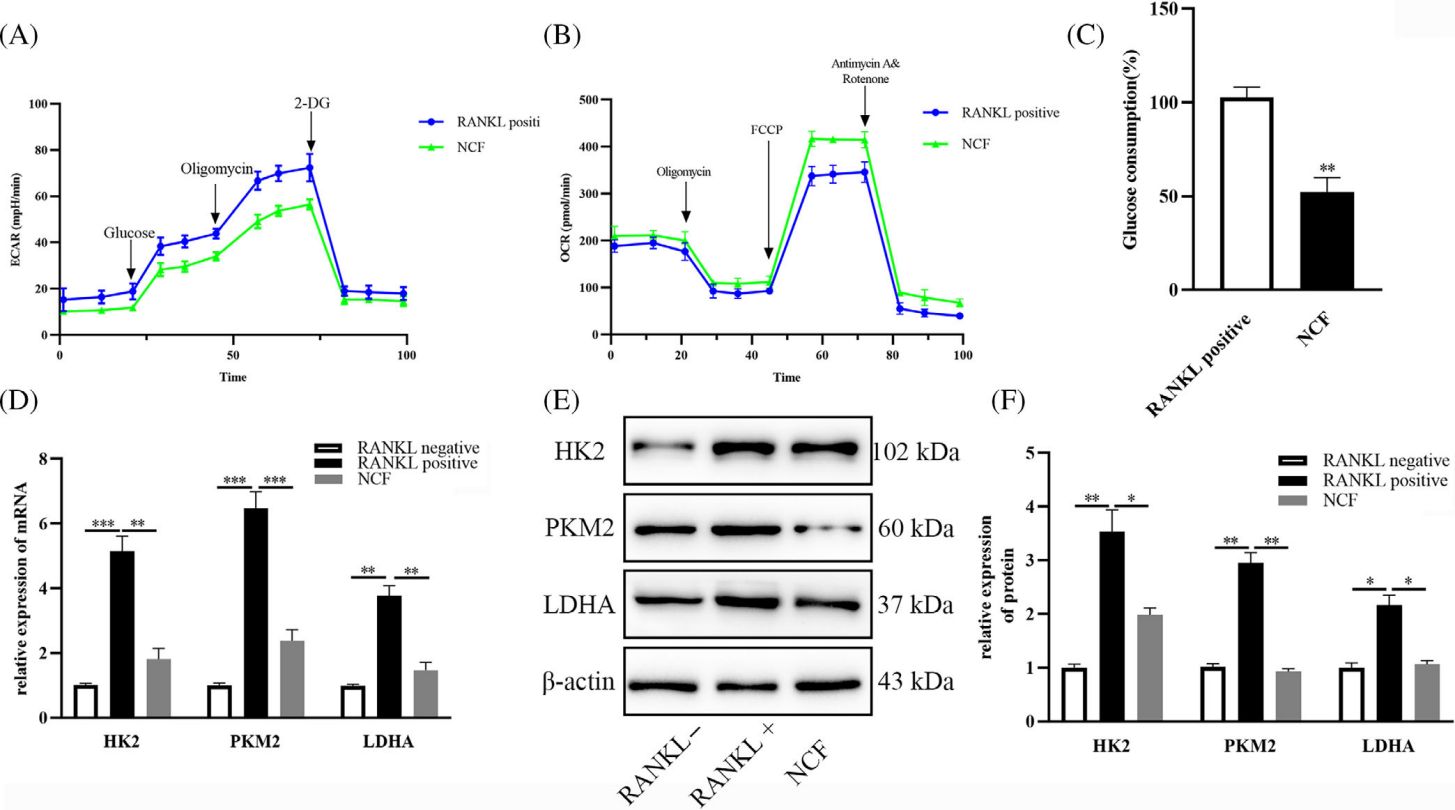
Nuciferine (NCF) disrupt glycolysis during, receptor activator of nuclear factor κb ligand (RANKL)‐induced osteoclastogenesis. (A) XF96 analyzer was used to detect extracellular acid rate. (B) XF96 analyzer was used to observe oxygen consumption. (C) Glucose consumption was detected to observe metabolism change. (D) RT‐PCR was performed to analyze the relative expression of glycolysis‐related mRNAs. (E) Western blot was performed to observe the expression of glycolysis‐related proteins. (F) Quantitative analysis of the expression of glycolysis‐related proteins (*p < 0.05; **p < 0.01; ***p < 0.001). HK2, hexokinase2; LDHA, lactate dehydrogenase A; OCR, oxygen consumption rate; PKM2, pyruvate kinase muscle 2.

It has been proved that glycolysis is an important process during osteoclast differentiation. To examine whether NCF disturbs metabolic programming in osteoclastogenesis, seahorse assay was performed. ECAR showed that the addition of NCF could lower the extracellular acid rate (Figure 2A), and oxygen consumption rate showed that NCF treatment could increase the oxygen consumption during RANKL‐induced osteoclast differentiation (Figure 2B). Besides, we could also observe that glucose consumption was decreased by NCF (Figure 2C). Then, the expression of key genes about glycolysis was detected. RT‐PCR and Western blot showed that the expressions of HK2, PKM2, and LDHA were all inhibited (Figure 2D–F). HK2, PKM2, and LDHA all participate in glycolysis and are rate‐limiting enzyme in glycolysis,^6^, ^7^, ^15^ which means that their expression could indicate the activity of glycolysis in osteoclast formation. These results showed that NCF could inhibit glycolysis.

### 3.3 Glycolysis inhibition is indispensable for NCF to hamper the formation of osteoclast

**FIGURE 3:**
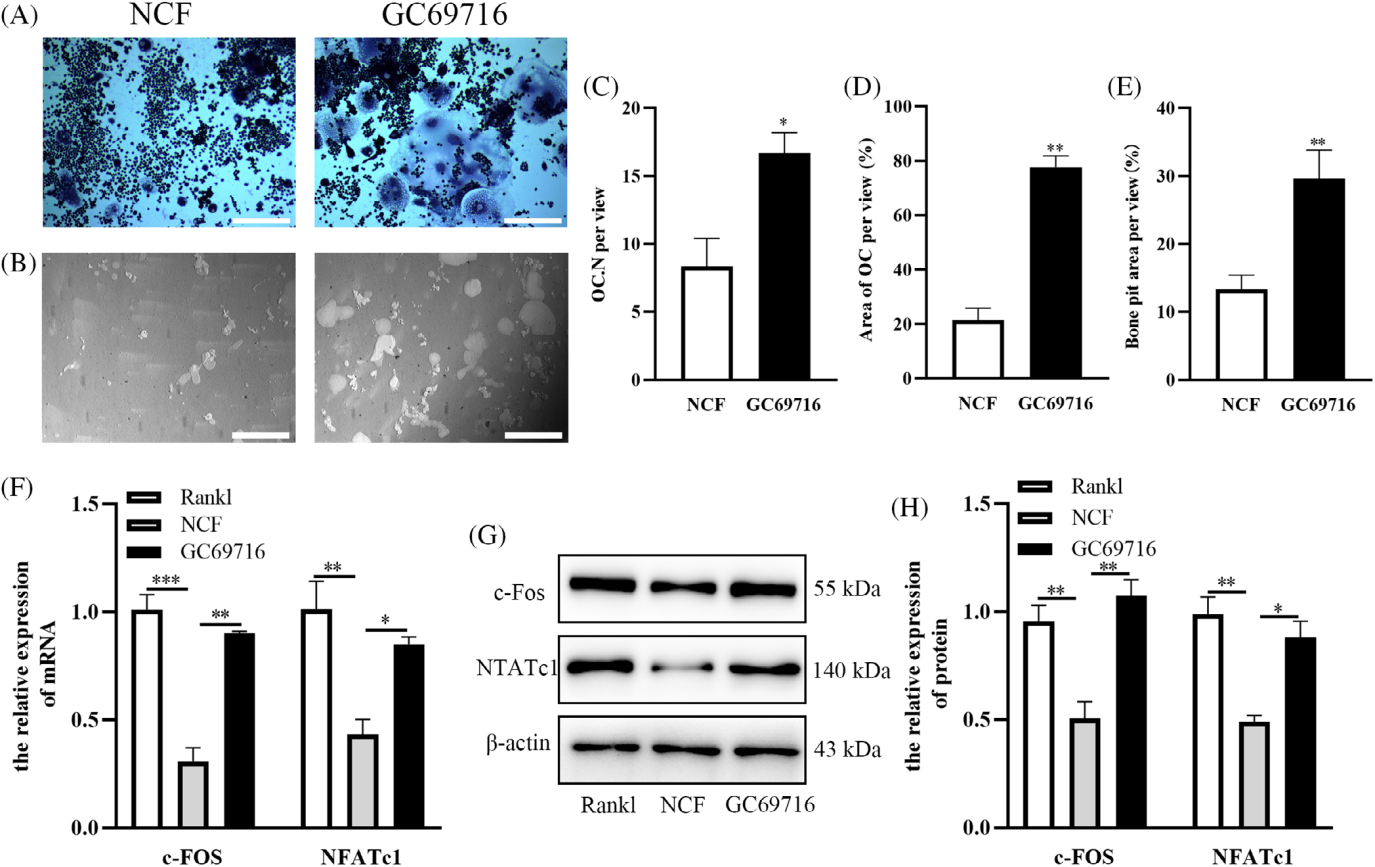
The inhibition of glycolysis is important for Nuciferine (NCF) to inhibit osteoclastogenesis. (A) Raw264.6 cells were induced into osteoclast differentiation with NCF treatment or with NCF + GC69716 (PKM2 activator) treatment, and trap staining was performed to observe osteoclasts. Scale bar = 200 μm (B) Bone resorption assay was performed to observe bone resorption. Scale bar = 400 μm (C) Quantitative analysis of osteoclast number per view. (D) Quantitative analysis of area covered by osteoclast per view. (E) Quantitative analysis of bone resorption area. (F) The relative expression of c‐Fos and nuclear factor of activated T cells c1 (NFATc1) mRNA was examined by RT‐PCR. (G) The relative expression of c‐Fos and NFATc1 protein was detected by Western blot. (H) Quantitative analysis of the expression of c‐Fos and NFATc1 protein (*p < 0.05; **p < 0.01; ***p < 0.001).

To determine whether glycolysis inhibition is necessary for NCF exerting its effects on osteoclast differentiation, 100 nM PKM2 activator (GC69716) was applied. When Raw264.7 cells were treated with NCF and GC69716 together, the formation of osteoclasts was recovered (Figure 3A). We could observe that the osteoclast number was increased by GC69716 (Figure 3C) and the area covered by osteoclast was also increased by GC69716 (Figure 3D). And bone resorption area also increased after GC69716 was added with NCF together (Figure 3B,E). Besides, RT‐PCR and Western blot demonstrated that the expression of c‐Fos and NFATc1 was promoted (Figure 3F–H). These data showed that the suppression of glycolysis was very important for NCF to inhibit the formation of osteoclast.

### 3.4 The decreased concentration of lactate might be responsible for NCF exerting its effects

**FIGURE 4:**
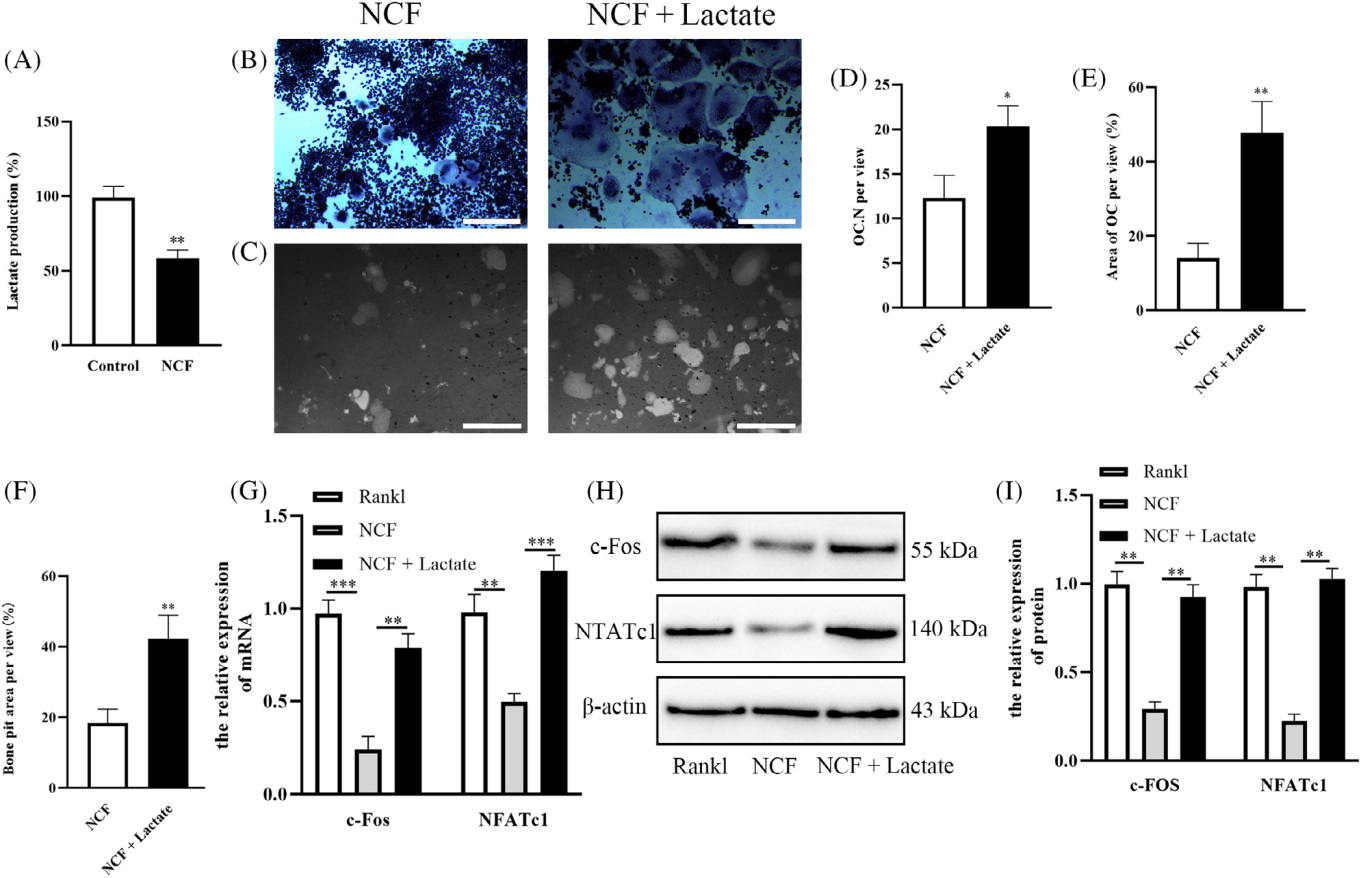
Lactate is decreased by Nuciferine (NCF) treatment and can restore NCF inhibited osteoclast formation. (A) Lactate concentration was detected when Raw264.7 cells were differentiated into osteoclast with or without NCF treatment. (B) Raw264.7 cells were induced into osteoclast with NCF treatment or with NCF + lactate treatment, and trap staining was used to observe osteoclast. Scale bar = 200 μm (C) Bone resorption assay was performed to observe bone resorption. Scale bar = 400 μm (D) Quantitative analysis of osteoclast number per view. (E) Quantitative analysis of area covered by osteoclast per view. (F) Quantitative analysis of bone resorption area. (G) The relative expression of c‐Fos and nuclear factor of activated T cells c1 (NFATc1) mRNA was examined by RT‐PCR. (H) The relative expression of c‐Fos and NFATc1 protein was detected by Western blot. (I) Quantitative analysis of the expression of c‐Fos and NFATc1 protein (*p < 0.05; **p < 0.01; ***p < 0.001). RANKL, receptor activator of nuclear factor κb ligand.

As an important product of glycolysis, lactate concentration was detected. The results showed that the concentration of lactate was decreased after the addition of NCF (Figure 4A). To determine whether lactate was needed for NCF to inhibit RANKL‐induced osteoclast differentiation, lactate was added with NCF together. Trap staining showed that lactate could recover the formation of osteoclast (Figure 4B); the increased number of osteoclast and area covered by osteoclast were all observed (Figure 4D,E). And bone resorption area was also increased after NCF addition (Figure 4C,F). And RT‐PCR and Western blot all showed that lactate addition could promote mRNA and protein expression of c‐Fos and NFATc1 (Figure 4G–I). These results demonstrated that lactate suppression might play an important role during NCF inhibiting osteoclastogenesis.

### 3.5 NCF suppresses increased ROS level caused by RANKL stimulation

**FIGURE 5:**
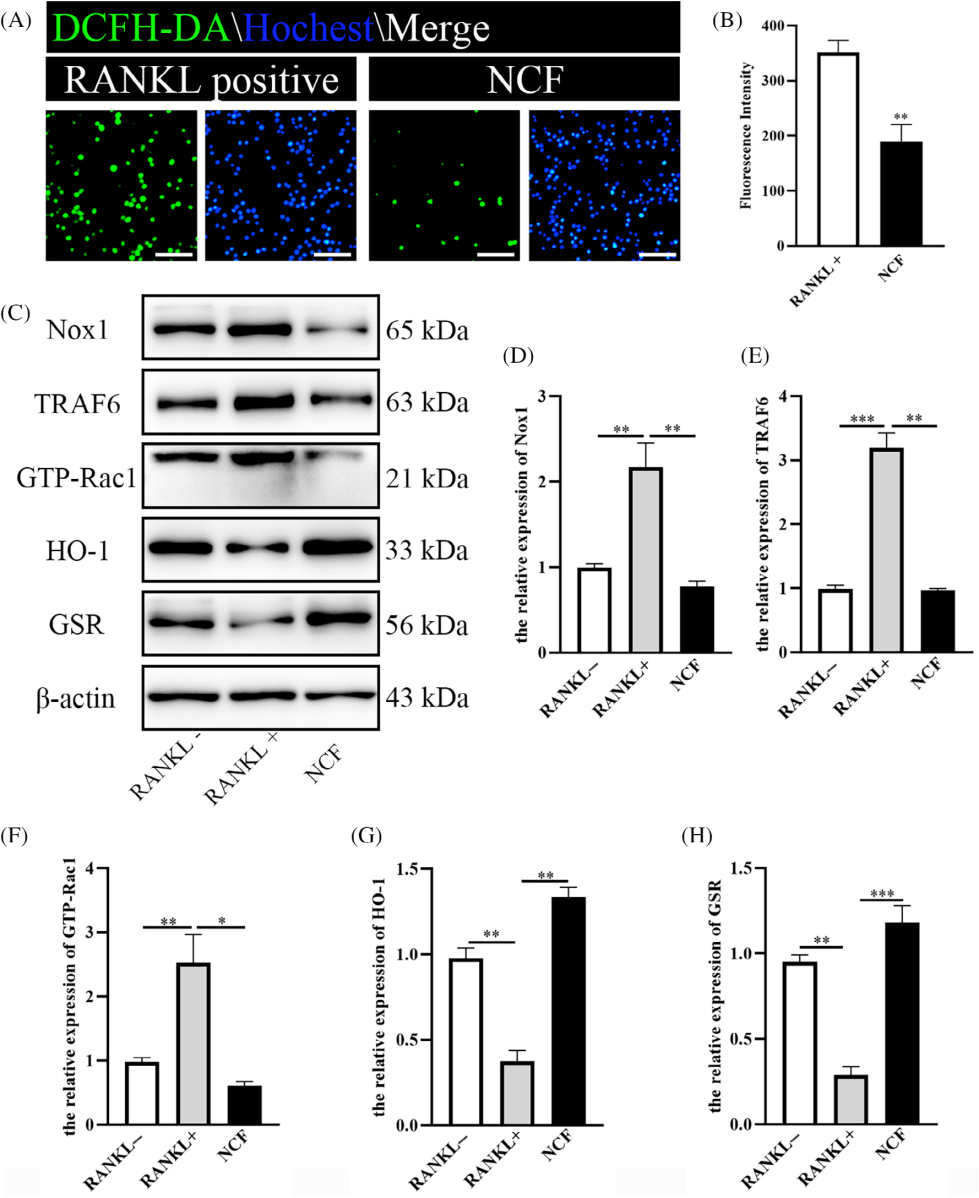
Nuciferine (NCF) inhibits the production of reactive oxygen species (ROS). (A) DCFH‐DA fluorescent probe was chosen to observe ROS level. Green indicated the fluorescent probes were activated by ROS. Scale bar = 50 μm (B) Quantitative analysis of fluorescence intensity. (C) Western blot was performed to detect the expression of NADPH Oxidase 1 (Nox1), TNF receptor‐associated factor 6 (TRAF6), Rac family small GTPase 1 (GTP‐Rac1), heme oxygenase 1 (HO‐1), and glutathione *S*‐reductase (GSR). (D–H) Quantitative analysis of the expression of Nox1, TRF6, GTP‐Rac1, HO‐1, and GSR (*p < 0.05; **p < 0.01; ***p < 0.001). RANKL, receptor activator of nuclear factor κb ligand.

In Figure 2B, NCF increases the proton leak, which is known to suppress ROS production and attenuate oxidative stress. Besides, NCF also decreased the production of lactate which has been reported to promote the production of ROS. These results suggested that NCF might suppress the production of ROS. To determine whether RANKL increased ROS was attenuated by NCF, ROS level was detected by oxidation‐sensitive dye DCFH‐DA and observed by fluorescence microscope. Results showed that NCF treatment significantly decreased the fluorescence intensity of DCFH‐DA, compared to the fluorescence intensity in Raw264.7 cells with RANKL stimulation only (Figure 5A,B). Then, we further explore the specific mechanism by which NCF inhibits ROS production. As the main source of ROS, Nox1 was examined. The protein level of Nox1 was increased by RANKL stimulation, but it was suppressed when NCF was added with RANKL together (Figure 5C,D). TRAF6 and Rac family small GTPase 1 (GTP‐Rac1) were indispensable for the activation of Nox1. Western blot results showed that the protein expression of TRAF6 and GTP‐Rac1 was upregulated by RANKL, and these trends were also reversed by the treatment of NCF (Figure 5C,E,F). And then, the expression of antioxidant enzymes like HO‐1 and GSR were also examined. These enzymes were inhibited by RANKL and were activated by the addition of NCF (Figure 5C,G,H). These results indicated that the antioxidant function enhanced the osteoclast inhibition effects of NCF.

## 4 DISCUSSION

Osteoclast is differentiated from monocyte/macrophage stimulated by RANKL. These cells are characterized with multi‐nucleus, positive TRAP staining, and bone resorption. During many skeletal diseases, such as osteoporosis and osteoarthritis, the relatively overactive function of osteoclast is the main cause of these disease. So, restriction of osteoclast function has been attracting much attention, and many drugs have been effective in treating these diseases based on this theory, such as denosumab and bisphosphonates. Although these anti‐osteoclast drugs can achieve a certain therapeutic effect, they also cause some unsatisfying side effects.^16^ So, in recent years, natural products have won the wide favor of researchers. In our previous research, NCF was uncovered to inhibit the formation of osteoclast and prevent the severity of osteoporosis.^4^ However, although we have demonstrated that NCF can suppress the activity of nuclear factor kappa B (NF‐κB) and mitogen‐activated protein kinase (MAPK) signaling pathways during the process of osteoclastogenesis, the deeper mechanism of NCF in the inhibition of osteoclast is still unclear. In this study, we discovered that NCF could inhibit RANKL‐induced glycolysis and exert antioxidant effects to inhibit the formation of osteoclast.

It has been demonstrated that glycolysis is indispensable for the formation of osteoclast.^6^, ^7^ Glycolysis, taking place in the cytoplasm, converts glucose into pyruvate with the production of ATP and NADPH. In the differentiation of mouse osteoclast, the expressions of several glycolysis‐related key genes are increased, such as glucose transporter type 1, HK2, phosphofructokinase (PFK), PKM2, and LDHA.^6^, ^7^, ^17^ Besides, the glucose consumption and the content of glycolysis byproduct‐lactate are also increased.^17^ In the present research, we discovered that the addition of NCF could inhibit the increased mRNA and protein expression of HK2, PKM2, and LDHA. And in the seahorse experiment, the oxygen consumption was increased and the ECAR was decreased when NCF was added with RANKL together. These results all indicated that the inhibition of glycolysis might be the key mechanism that NCF inhibits the RANKL‐induced differentiation of osteoclast.

In the past research, we have reported that NCF could suppress the osteoclast formation through the suppression of c‐Fos and NFATc1 via inhibiting the upper signaling pathways of NF‐κB and MAPK signaling pathways. NF‐κB and MAPK signaling pathway has been reported to be the key pathway in regulating the differentiation of osteoclast.^18^, ^19^, ^20^, ^21^ However, some researches also reported that NF‐κB organizes the balance of glycolysis and OXPHOS.^22^ To realize the multi‐effects of differentiation, proliferation, survival, and metabolic regulation, the NF‐κB signaling pathway covers five different DNA‐binding subunits containing N‐terminal NF‐κB /Rel homology domain, namely RELA (p65), RELB, and REL (c‐Rel), NF‐κB2 (p100/p52) and NF‐κB1 (p105/p50).^22^ In immunotolerant macrophages, the NF‐κB signaling pathway mediates the glycolysis regulated by NLR family CARD domain containing 3.^23^ The binding failure of p65 and nuclear factor of activated T cells 5 induced by NLR family CARD domain containing 3 resulted in the decreased expression of glycolytic genes, such as glucose transporter type 1, HK2, PFKFB3, and PFK.^23^ In some other researches, NF‐κB signaling pathway could facilitate glycolysis though enhancing the activation of hypoxia inducible factor 1 subunit α.^24^ However, other researchers also demonstrated that glycolysis could also regulate the activation of the NF‐κB signaling pathway. Yating Wang found that PFKFB3‐mediated glycolysis reprogramming could promote the expression of NF‐κB signaling genes through enhanced histone lactylation.^25^ So, we speculated that glycolysis might be the key process in the interruption of osteoclast formation mediated by the NF‐κB signaling pathway. In addition to the NF‐κB signaling pathway, the MAPK signaling pathway also participates in the process of glycolysis. The P38 MAPK signaling pathway plays a key role in the glycolysis of gastric cancer cells.^26^ Yang et al.^27^ also found that the ERK MAPK signaling pathway could up regulate the expression of HK1, HK2, and LDHA, and subsequently, activate the glycolytic metabolism. Therefore, combined with the analysis of previous research results, we speculate that the inhibition of glycolysis may be the key downstream cellular activity of NCF to inhibit osteoclast formation.

As it has been widely accepted that NCF has wide antioxidant effects,^28^ we also discovered that NCF could suppress oxidative stress induced by RANKL stimulation. In the present research, we found that ROS level was increased after the stimulation of RANKL and was decreased with the treatment of NCF, which mean that NCF could exert its antioxidant effect in osteoclast formation. ROS is an important signaling messenger in the formation of osteoclast.^29^ Once RANK is stimulated by RANKL, ROS production is activated by TRAF6, and ROS level would be balanced by the activation of Nox‐1, HO‐1, and GSR.^30^, ^31^ Nox‐1 is one of the main factors that determine the level of ROS,^32^ and its activation depends on TRAF6 and Rac1.^30^ We found that NCF could decrease the expression of TRAF6 and Rac1 which were increased by RANKL stimulation, and as expected, the expression of Nox‐1 was decreased after the addition of NCF followingly. Besides, we also detected the effects of NCF on the antioxidant enzymes such as HO‐1 and GSR. The results demonstrated that NCF could also promote the expression of HO‐1 and GSR. These results indicated that while regulating the formation of osteoclast, NCF could not only suppress the expression of oxidase but also strengthen the expression of antioxidant enzymes. As it has been reported, ROS is an important activator of NF‐ κB and MAPK signaling pathways.^21^, ^33^, ^34^ These results prompt us that ROS attenuation might be the upper stream mechanism that NCF inhibits the activation of NF‐κB and MAPK signaling pathways. Interestingly, ROS could also inhibit osteogenic differentiation of bone marrow mesenchymal stem cells and scavenging ROS could promote osteogenesis.^35^ Bharathi et al.^36^ reported that the addition of NCF in scaffold strengthened the osteogenic potential of mouse mesenchymal stem cells through promoting the expression of Runx2, alkaline phosphatase, type I collagen, and Osteocalcin. Although this research had not explored the effects of NCF on osteogenesis, these results also indicate that antioxidant effect might be the key mechanism by which NCF promotes osteogenesis. Furthermore, the activation of NF‐κB and MAPK signaling pathways and the increase of intercellular ROS level all promote the dysfunction of endothelial cells which also play fundamental effects during osteogenesis.^37^, ^38^ We speculate that NCF could promote angiogenesis which is suppressed during osteoporosis. So, we consider that the prevention and treatment of NCF on osteoporosis may be due to the inhibited osteoclastogenesis, promoted osteogenesis, and strengthened angiogenesis.

In conclusion, this research showed that NCF could reprogram the glucose metabolism and inhibit glycolysis during RANKL‐induced osteoclastogenesis. And as one of the antioxidant products, NCF could also exert antioxidant effects in osteoclastogenesis. Based on these results and previous results that NCF could inhibit osteoclast formation through inactivating NF‐κB and MAPK signaling pathways, we speculate that the decreased level of ROS might be the key factor mediating the inactivation of NF‐κB and MAPK signaling pathways by NCF, and NCF might inhibit osteoclast formation through suppression of glycolysis via the inactivation of NF‐κB and MAPK signaling pathways. Compared with other anti‐osteoporosis drugs, NCF has a wider range of biological activities, such as anti‐inflammatory, anti‐obesity, and anti‐diabetes,^39^ which all contribute to the prevention of osteoporosis. In addition, NCF has a wide range of sources and a wider history of medication and might be more acceptable to patients. However, in the research, the used concentration of 30 μM might limit the further application of NCF in anti‐osteoporosis. Besides, poor absorption, rapid metabolism, and rapid systemic elimination of NCF result in its poor bioavailability which may hamper the clinical application of NCF.^40^ Therefore, how to improve the bioavailability of NCF may be a key factor restricting the clinical application of NCF against osteoporosis, and this will also be one of the key points of our future work.

## CONFLICT OF INTEREST STATEMENT

The authors declare no conflict of interest.

## References

[1] Föger‐Samwald U , Dovjak P , Azizi‐Semrad U , Kerschan‐Schindl K , Pietschmann P . Osteoporosis: pathophysiology and therapeutic options. EXCLI J. 2020;19:1017–1037. 32788914 10.17179/excli2020-2591 PMC7415937

[2] Wu Y , Ai H , Xi Y , Yin P , Qu Y , Xu J , et al. Reduced osteoclast‐derived apoptotic bodies in bone marrow characterizes the pathological progression of osteoporosis. Cell Death Discov. 2023;9(1):135. 37185334 10.1038/s41420-023-01434-w PMC10130088

[3] Zhang P , Chen H , Xie B , Zhao W , Shang Q , He J , et al. Bioinformatics identification and experimental validation of m6A‐related diagnostic biomarkers in the subtype classification of blood monocytes from postmenopausal osteoporosis patients. Front Endocrinol (Lausanne). 2023;14:990078. 36967763 10.3389/fendo.2023.990078 PMC10031099

[4] Song C , Cao J , Lei Y , Chi H , Kong P , Chen G , et al. Nuciferine prevents bone loss by disrupting multinucleated osteoclast formation and promoting type H vessel formation. FASEB J. 2020;34(3):4798–4811. 32039519 10.1096/fj.201902551R

[5] Takegahara N , Kim H , Choi Y . Unraveling the intricacies of osteoclast differentiation and maturation: insight into novel therapeutic strategies for bone‐destructive diseases. Exp Mol Med. 2024;56(2):264–272. 38297158 10.1038/s12276-024-01157-7 PMC10907717

[6] Nishioku T , Anzai R , Hiramatsu S , Terazono A , Nakao M , Moriyama M . Lactate dehydrogenase a inhibition prevents RANKL‐induced osteoclastogenesis by reducing enhanced glycolysis. J Pharmacol Sci. 2023;153(4):197–207. 37973217 10.1016/j.jphs.2023.09.005

[7] Li F , Liu X , Li M , Wu S , Le Y , Tan J , et al. Inhibition of PKM2 suppresses osteoclastogenesis and alleviates bone loss in mouse periodontitis. Int Immunopharmacol. 2024;129:111658. 38359663 10.1016/j.intimp.2024.111658

[8] Ahn H , Lee K , Kim JM , Kwon SH , Lee SH , Lee SY , et al. Accelerated lactate dehydrogenase activity potentiates osteoclastogenesis via NFATc1 signaling. PLoS One. 2016;11(4):e0153886. 27077737 10.1371/journal.pone.0153886 PMC4831772

[9] Gong H , Zhong H , Cheng L , Li LP , Zhang DK . Post‐translational protein lactylation modification in health and diseases: a double‐edged sword. J Transl Med. 2024;22(1):41. 38200523 10.1186/s12967-023-04842-9 PMC10777551

[10] Qian J , Gong ZC , Zhang YN , Wu HH , Zhao J , Wang LT , et al. Lactic acid promotes metastatic niche formation in bone metastasis of colorectal cancer. Cell Commun Signal. 2021;19(1):9. 33478523 10.1186/s12964-020-00667-x PMC7818572

[11] Taubmann J , Krishnacoumar B , Bohm C , Faas M , Muller DIH , Adam S , et al. Metabolic reprogramming of osteoclasts represents a therapeutic target during the treatment of osteoporosis. Sci Rep. 2020;10(1):21020. 33273570 10.1038/s41598-020-77892-4 PMC7713370

[12] Cheng C , Li W , Ye Y , Zhu Y , Tang M , Hu Z , et al. Lactate induces C2C12 myoblasts differentiation by mediating ROS/p38 MAPK signalling pathway. Tissue Cell. 2024;87:102324. 38354685 10.1016/j.tice.2024.102324

[13] Yan C , Zhan Y , Yuan S , Cao Y , Chen Y , Dong M , et al. Nuciferine prevents obesity by activating brown adipose tissue. Food Funct. 2024;15(2):967–976. 38175708 10.1039/d3fo03632d

[14] Song C , Yang X , Lei Y , Zhang Z , Smith W , Yan J , et al. Evaluation of efficacy on RANKL induced osteoclast from RAW264.7 cells. J Cell Physiol. 2019;234(7):11969–11975. 30515780 10.1002/jcp.27852

[15] Zhang L , Jiang C , Zhong Y , Sun K , Jing H , Song J , et al. STING is a cell‐intrinsic metabolic checkpoint restricting aerobic glycolysis by targeting HK2. Nat Cell Biol. 2023;25(8):1208–1222. 37443289 10.1038/s41556-023-01185-x PMC11232535

[16] Zhao Q , Feng J , Liu F , Liang Q , Xie M , Dong J , et al. Rhizoma Drynariae‐derived nanovesicles reverse osteoporosis by potentiating osteogenic differentiation of human bone marrow mesenchymal stem cells via targeting ERalpha signaling. Acta Pharm Sin B. 2024;14(5):2210–2227. 38799625 10.1016/j.apsb.2024.02.005 PMC11119514

[17] Ledesma‐Colunga MG , Passin V , Lademann F , Hofbauer LC , Rauner M . Novel insights into osteoclast energy metabolism. Curr Osteoporos Rep. 2023;21(6):660–669. 37816910 10.1007/s11914-023-00825-3 PMC10724336

[18] Ji H , Pan Q , Cao R , Li Y , Yang Y , Chen S , et al. Garcinone C attenuates RANKL‐induced osteoclast differentiation and oxidative stress by activating Nrf2/HO‐1 and inhibiting the NF‐kB signaling pathway. Heliyon. 2024;10(3):e25601. 38333852 10.1016/j.heliyon.2024.e25601 PMC10850749

[19] Zheng T , Lin Z , Jiang G , Chen H , Yang Y , Zeng X . Pogostone attenuates osteolysis in breast cancer by inhibiting the NF‐kB and JNK signaling pathways of osteoclast. Life Sci. 2023;328:121611. 37068706 10.1016/j.lfs.2023.121611

[20] Wang J , Chen G , Yang X , Dou W , Mao Y , Zhang Y , et al. Inhibitory effects of norcantharidin on titanium particle‐induced osteolysis, osteoclast activation and bone resorption via MAPK pathways. Int Immunopharmacol. 2024;129:111655. 38340423 10.1016/j.intimp.2024.111655

[21] Jin C , Yu XB , Yang J , Lin Z , Ma RX , Lin BH , et al. Corynoline suppresses Osteoclastogenesis and attenuates ROS activities by regulating NF‐kappaB/MAPKs and Nrf2 signaling pathways. J Agric Food Chem. 2024;72(14):8149–8166. 38551844 10.1021/acs.jafc.3c07088

[22] Kracht M , Müller‐Ladner U , Schmitz ML . Mutual regulation of metabolic processes and proinflammatory NF‐κB signaling. J Allergy Clin Immunol. 2020;146(4):694–705. 32771559 10.1016/j.jaci.2020.07.027

[23] Xu J , Gao C , He Y , Fang X , Sun D , Peng Z , et al. NLRC3 expression in macrophage impairs glycolysis and host immune defense by modulating the NF‐κB‐NFAT5 complex during septic immunosuppression. Mol Ther. 2023;31(1):154–173. 36068919 10.1016/j.ymthe.2022.08.023 PMC9840117

[24] Zhang Y , Yang N , Li Y , Tan C , Cai Y , Rui X , et al. Transmissible gastroenteritis virus induces inflammatory responses via RIG‐I/NF‐κB/HIF‐1α/glycolysis axis in intestinal organoids and in vivo. J Virol. 2024;98(6):e0046124. 38780247 10.1128/jvi.00461-24 PMC11237398

[25] Wang Y , Li H , Jiang S , Fu D , Lu X , Lu M , et al. The glycolytic enzyme PFKFB3 drives kidney fibrosis through promoting histone lactylation‐mediated NF‐kappaB family activation. Kidney Int. 2024;106(2):226–240. 38789037 10.1016/j.kint.2024.04.016

[26] He J , Yi J , Ji L , Dai L , Chen Y , Xue W . ECHDC2 inhibits the proliferation of gastric cancer cells by binding with NEDD4 to degrade MCCC2 and reduce aerobic glycolysis. Mol Med. 2024;30(1):69. 38783226 10.1186/s10020-024-00832-9 PMC11118108

[27] Yang S , Tang W , Azizian A , Gaedcke J , Ohara Y , Cawley H , et al. MIF/NR3C2 Axis regulates glucose metabolism reprogramming in pancreatic cancer through MAPK‐ERK and AP‐1 pathways. Carcinogenesis. 2024;45(8):582–594. 38629149 10.1093/carcin/bgae025 PMC11317528

[28] Zhou Z , Qi J , Wu Y , Li C , Bao W , Lin X , et al. Nuciferine effectively protects mice against acetaminophen‐induced liver injury. Antioxidants (Basel). 2023;12(4):949. 37107324 10.3390/antiox12040949 PMC10136285

[29] Liu Z , Gao Y , Feng X , Su Y , Lian H , Zhao J , et al. Hecogenin alleviates LPS‐induced osteolysis via regulating pyroptosis and ROS involved Nrf2 activation. Biomed Pharmacother. 2024;177:116933. 38901204 10.1016/j.biopha.2024.116933

[30] Chen K , Qiu P , Yuan Y , Zheng L , He J , Wang C , et al. Pseurotin a inhibits osteoclastogenesis and prevents ovariectomized‐induced bone loss by suppressing reactive oxygen species. Theranostics. 2019;9(6):1634–1650. 31037128 10.7150/thno.30206 PMC6485188

[31] Lin X , Yuan G , Yang B , Xie C , Zhou Z , Liu Y , et al. Dauricine attenuates ovariectomized‐induced bone loss and RANKL‐induced osteoclastogenesis via inhibiting ROS‐mediated NF‐kappaB and NFATc1 activity. Phytomedicine. 2024;129:155559. 38579642 10.1016/j.phymed.2024.155559

[32] Lian S , Wang T , Li J , Yang Q , Lu C . Tauroursodeoxycholic acid mitigates oxidative stress and promotes differentiation in high salt‐stimulated osteoblasts via NOX1 mediated by PGC‐1alpha. Discov Med. 2024;36(183):788–798. 38665027 10.24976/Discov.Med.202436183.74

[33] Asiwe JN , Ajayi AM , Ben‐Azu B , Fasanmade AA . Vincristine attenuates isoprenaline‐induced cardiac hypertrophy in male Wistar rats via suppression of ROS/NO/NF‐қB signalling pathways. Microvasc Res. 2024;155:104710. 10.1016/j.mvr.2024.104710 38880384

[34] Hu Y , Hu H , Yin L , Wang L , Luo K , Luo N . Arachidonic acid impairs the function of the blood‐testis barrier via triggering mitochondrial complex‐ROS‐P38 MAPK axis in hyperthermal Sertoli cells. Ecotoxicol Environ Saf. 2023;252:114598. 36774800 10.1016/j.ecoenv.2023.114598

[35] Zhang Q , Chen W , Li G , Ma Z , Zhu M , Gao Q , et al. A factor‐free hydrogel with ROS scavenging and responsive degradation for enhanced diabetic bone healing. Small. 2024;20(24):e2306389. 38168513 10.1002/smll.202306389

[36] Bharathi R , Harini G , Sankaranarayanan A , Shanmugavadivu A , Vairamani M , Selvamurugan N . Nuciferine‐loaded chitosan hydrogel‐integrated 3D‐printed polylactic acid scaffolds for bone tissue engineering: a combinatorial approach. Int J Biol Macromol. 2023;253(Pt 7):127492. 37858655 10.1016/j.ijbiomac.2023.127492

[37] Kusumbe AP , Ramasamy SK , Adams RH . Coupling of angiogenesis and osteogenesis by a specific vessel subtype in bone. Nature. 2014;507(7492):323–328. 24646994 10.1038/nature13145 PMC4943525

[38] Lee CY , Wu SW , Yang JJ , Chen WY , Chen CJ , Chen HH , et al. Vascular endothelial dysfunction induced by 3‐bromofluoranthene via MAPK‐mediated‐NFκB pro‐inflammatory pathway and intracellular ROS generation. Arch Toxicol. 2024;98(7):2247–2259. 38635053 10.1007/s00204-024-03751-0 PMC11169047

[39] Wu X‐L , Wu M‐J , Chen X‐Z , Ma H‐L , Ding L‐Q , Qiu F , et al. Metabolic profiling of nuciferine in rat urine, plasma, bile and feces after oral administration using ultra‐high performance liquid chromatography‐diode array detection‐quadrupole time‐of‐flight mass spectrometry. J Pharm Biomed Anal. 2017;140:71–80. 28342305 10.1016/j.jpba.2017.03.022

[40] Liu Y , Wu X , Mi Y , Zhang B , Gu S , Liu G , et al. PLGA nanoparticles for the oral delivery of nuciferine: preparation, physicochemical characterization and in vitro/in vivo studies. Drug Deliv. 2017;24(1):443–451. 28165858 10.1080/10717544.2016.1261381 PMC8241190

